# Various Aspects of a Gene Editing System—CRISPR–Cas9

**DOI:** 10.3390/ijms21249604

**Published:** 2020-12-16

**Authors:** Edyta Janik, Marcin Niemcewicz, Michal Ceremuga, Lukasz Krzowski, Joanna Saluk-Bijak, Michal Bijak

**Affiliations:** 1 Biohazard Prevention Centre, Faculty of Biology and Environmental Protection, University of Lodz, Pomorska 141/143, 90-236 Lodz, Poland; edyta.janik@unilodz.eu (E.J.); marcin.niemcewicz@biol.uni.lodz.pl (M.N.); 2 Military Institute of Armament Technology, Prymasa Stefana Wyszyńskiego 7, 05-220 Zielonka, Poland; ceremugam@witu.mil.pl; 3 Biodefense Laboratory, Biomedical Engineering Centre, Institute of Optoelectronics, Military University of Technology, gen. Sylwestra Kaliskiego 2, 00-908 Warsaw, Poland; lukasz.krzowski@wat.edu.pl; 4 Department of General Biochemistry, Faculty of Biology and Environmental Protection, University of Lodz, Pomorska 141/143, 90-236 Lodz, Poland; joanna.saluk@biol.uni.lodz.pl

**Keywords:** CRISPR–Cas9, gene editing, prime editing

## Abstract

The discovery of clustered, regularly interspaced short palindromic repeats (CRISPR) and their cooperation with CRISPR-associated (Cas) genes is one of the greatest advances of the century and has marked their application as a powerful genome engineering tool. The CRISPR–Cas system was discovered as a part of the adaptive immune system in bacteria and archaea to defend from plasmids and phages. CRISPR has been found to be an advanced alternative to zinc-finger nucleases (ZFN) and transcription activator-like effector nucleases (TALEN) for gene editing and regulation, as the CRISPR–Cas9 protein remains the same for various gene targets and just a short guide RNA sequence needs to be altered to redirect the site-specific cleavage. Due to its high efficiency and precision, the Cas9 protein derived from the type II CRISPR system has been found to have applications in many fields of science. Although CRISPR–Cas9 allows easy genome editing and has a number of benefits, we should not ignore the important ethical and biosafety issues. Moreover, any tool that has great potential and offers significant capabilities carries a level of risk of being used for non-legal purposes. In this review, we present a brief history and mechanism of the CRISPR–Cas9 system. We also describe on the applications of this technology in gene regulation and genome editing; the treatment of cancer and other diseases; and limitations and concerns of the use of CRISPR–Cas9.

## 1. Introduction

Precise and efficient genome modification is significant for genetic engineering. The progressive development of technology enables the use of techniques that allow for changes in genomes. The most essential tools for genome editing are enzymes, such as transcription activator-like effector nucleases (TALENs) and zinc finger nucleases (ZFNs) [1]. Genome editing tools are determined by a common feature. They can all be designed to introduce DNA double-strand breaks (DSBs) at desired locations in the genome. A DSB activates the cell’s natural DNA repair machinery, which can be used to improve the efficiency of introducing alterations into the genome [2]. The discovery of clustered regularly-interspaced short palindromic repeats (CRISPR); their description as an adaptative prokaryotic immune system (CRISPR–Cas), providing specific and acquired immunity against mobile and exogenic genetic elements; and the following development into a precision genomic editing tool has changed the field of molecular biology [3,4]. CRISPRs were identified in the *Escherichia coli* genome in 1987, and they were characterized as extraordinary sequence elements, which consisted of a series of 29 nucleotide repeats separated by 32 nucleotide “spacer” sequences, which appeared whenever bacteria came in contact with phage DNA [5]. At the time, scientists could not predict the biological functions of those uncommon sequences because of the lack of adequate DNA sequence information, in particular for mobile genetic elements [6]. In 1993, CRISPRs were observed in archaea, specifically in *Haloferax mediterranei* [7]. Then, CRISPRs were recognized in phylogenetically diverse archaeal and bacterial genomes, and four genes regularly present adjacent to the CRISPR regions were discovered. It was considered that the genes were related to CRISPR and were defined as CRISPR-associated genes 1 through 4 (*cas1* to *cas4*) [8,9,10]. CRISPR loci are now observed in approximately 84% of archaeal genomes and in 45% of bacterial genomes [11]. Next, Haft et al. described 41 new *cas* gene families present near CRISPR, in addition to the four previously discovered. Two of the 45 *cas* genes (*cas1* and *cas2*) are present in all families and are also involved in spacer acquisition. All those studies and analyses have shown that CRISPR systems belong to various classes, with diverse repeat patterns, sets of genes and ranges of species [12,13]. Subsequent comparative genomic analysis indicated that CRISPR and Cas proteins cooperate and provide an acquired immune system to protect prokaryotic cells from invading genetic elements, such as viruses and plasmids, analogous to the eukaryotic RNA interference (RNAi) system. This assumption was experimentally proven in 2007, using the lactic acid bacteria *Streptococcus thermophilus* [3]. The molecular mechanism of the adaptive immune response to a phage infection was explained in 2007 [3]. It was demonstrated that CRISPRs are transcribed into RNA, which is next cleaved and loaded into CRISPR–Cas proteins, and the RNA–protein complex is sufficient for RNA-guided dsDNA endonuclease activity [14,15].

## 2. Classification

CRISPR Cas systems are divided into two major classes, six types and 33 subtypes. The overall classifications and characteristics are summarized in Table 1. The CRISPR–Cas system class 1 contains multiprotein effector complexes. Class 2 is defined by a single, multidomain, multifunctional effector protein [16]. Those classes are divided into three types each. In class 1, there are I, III and IV types, and class 2 includes II, V and VI types.

All types are distinguished by different architectures of the effector modules, which contain unique signature proteins. Each type is also classified into numerous subtypes that are characterized by subtle differences in locus organization and encode subtype-specific Cas proteins [12,17,21]. The primary features that define the type and subtype of CRISPR–Cas systems are *cas* genes and the proteins they encode, which are genetically and functionally diverse. That illustrates the number of biochemical functions that they perform at various steps of CRISPR-mediated immunity. The RNA recognition motif (RRM) is prevalent in numerous Cas proteins and many of the Cas proteins’ families contain functional domains which interact with nucleic acids, helicase and nuclease motifs [22,23]. Genetically, *cas1* and *cas2* are commonly present in different types and subtypes, while signature genes such as *cas3*, *cas9* and *cas10* have been determined for types I, II and III, respectively [24].

## 3. Mechanism of Action

**Figure 1:**
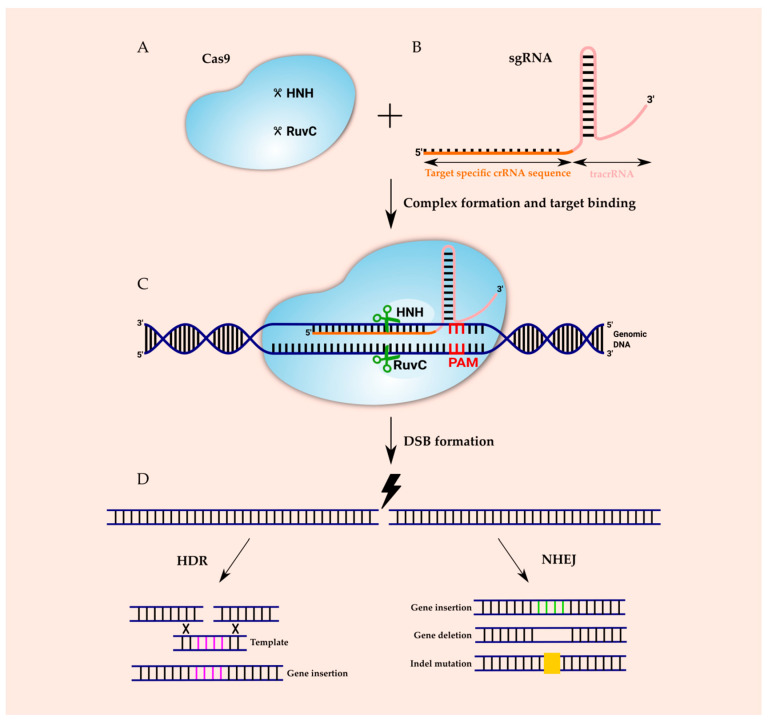
Schematic diagram of the CRISPR–Cas 9 system molecular mechanism. The CRISPR/Cas 9 system is composed of sgRNA and Cas9. The Cas9 protein contains two nuclease domains—the RuvC domain, which cleaves non-complementary DNA strands, and the HNH domain, which cleaves complementary DNA strands (**A**). The sgRNA includes the trans-activating crispr RNA (tracrRNA) and crispr RNA (crRNA). The crRNA comprises a 20 nt protospacer element and a few additional nucleotides, which are complementary to the tracrRNA (**B**). The tracrRNA hybridizes to the crRNA and binds to the Cas9 protein forming the CRISPR–Cas9/sgRNA complex in order to edit the genome sequences. The Cas9–sgRNA complex unwinds the dsDNA, and the complementary sequence in sgRNA anneals to one of DNA strands. Upon binding, the endonuclease domains cleave both DNA strands three bases upstream of the protospacer-adjacent motif (PAM) sequence (**C**). The double-strand break (DSB) in DNA forms and then is repaired either by a homology directed repair (HDR) pathway, if an appropriate donor is present, or by non-homologous end joining (NHEJ). HDR repair allows precise genome editing at the target site, and NHEJ introduces short insertions, deletions or indels (**D**).

The CRISPR–Cas immune response includes three steps: adaptation, expression and interference. In the adaptation step, a complex of Cas proteins encounters a short protospacer-adjacent motif (PAM), binds to an invading DNA molecule and causes two double-strand breaks in it. The released short DNA fragment of invading phages or plasmids (termed protospacer) is integrated between two repeats of CRISPR array and becomes a spacer. In the expression stage, *cas* genes’ expression and transcription of the CRISPR into a long precursor CRISPR RNA (pre-crRNA) occurs. Cas proteins and accessory factors process pre-crRNA into short mature crRNA. In the interference step, the combined action of crRNA and Cas proteins recognizes and mediates the cleavage of the foreign nucleic acid, consequently protecting the host cells from the infection [3,25]. The expression and interference stages are different in each of the CRISPR systems. In type I, Cas6e/Cas6f cut at the junction of double-stranded RNA (dsRNA) and single-stranded RNA (ssRNA) that has been formed by hairpin loops. In the type II system, transactivating crRNAs (tracrRNAs) are involved to form dsRNA, cleaved by Cas9 and RNase III. In type III, a Cas6 homolog is used in the direct repeat for cleavage and hairpin loops are not required [14,26]. The CRISPR can be found on both plasmid and chromosomal DNA. The lengths of spacers and the lengths and sequences of repeats are well conserved within a CRISPR locus; however, they may differ between CRISPRs in the same or other genomes. CRISPR repeats can differ (23–55 nt), although repeats typically have lengths of 28–37 nt and each contain a palindromic sequence that can form hairpin structures. Similarly, spacers can vary widely (21–72 nt), but their typical length is 32–38 nt [11]. As mentioned above, numerous Cas proteins bind to nucleic acids, making the CRISPR system a tool for genomic engineering. Amongst the Cas proteins, Cas 9 is the most commonly used for genomic editing and regulation [27]. The molecular mechanism of the CRISPR–Cas9 system-mediated genome-editing is illustrated in Figure 1. Cas9 is comprised of two nuclease domains: an HNH (His–Asn–His) nuclease which cleaves the target strand of DNA, and RuvC-like nuclease, which splits into RuvC-I, RuvC-II and RuvC-III subdomains and cleaves the nontarget strand. Cas9 forms a ribonucleoprotein complex with two RNAs: the crRNA which recognizes the foreign DNA and the tracrRNA that hybridizes with crRNA and is distinctive for the type II. For efficient genomic editing, the crRNA and tracrRNA can be fused into a chimeric single-guide RNA (sgRNA) [14,15]. sgRNA is composed of two parts: a constant part that forms a scaffold for Cas9 binding and the 5′-end, and a 20 nt part which is complementary to target DNA sequence.

The target site in DNA also includes two parts: the protospacer complementary to the 5′-end and 20 nt sequence in sgRNA, and a short PAM bound by Cas9 which is directly adjacent to the protospacer. sgRNA recognizes a specific sequence in the genome, and Cas9 acts as a pair of scissors to cleave the DNA sequence. The Cas9 will not cleave sequence in the absence of a PAM. Different bacterial type II CRISPR systems have different Cas9 proteins. The most commonly used Cas9 has been adapted from *Streptococcus pyogenes* (SpCas9) and identifies the 5′-NGG-3′ sequence on the non-target DNA strand as the PAM [28,29]. The consequence of pairing the protospacer with the 5′-end 20 nt sequence and the binding of Cas9 to PAM is the formation of a DSB which triggers DNA repair [30]. There are two major endogenous repair mechanisms in eukaryotes: non-homologous end joining (NHEJ) and homology directed repair (HDR) [2]. In the NHEJ mechanism, protein factors rejoin DNA strands directly or do so by including nucleotide deletions or insertions. Nevertheless, this repair mechanism is an error-prone process which can result in the semirandom deletion or addition of DNA base pairs. What is more, NHEJ can initiate frameshift mutations into the targeted gene and disrupt it [31,32,33]. NHEJ can occur at any phase of the cell cycle. On the contrary, HDR occurrence is limited to late S or G_2_ phase, when sister chromatids are available and can serve as repair temples. The HDR mechanism requires the presence of homologous donor DNA sequences from sister chromatids or foreign DNA to result in precise insertions and base substitutions between two DSBs or DSB sites [34,35].

## 4. Comparison to ZFNs and TALENs

Technological progress is essential for innovative biological research. The developments of molecular tools for DNA manipulation, such as ZFN, TALEN and CRISPR–Cas, have revolutionized genome editing [36]. Table 2 shows a systematic comparison of the three platforms. Compared with previous programmable gene editing tools, CRISPR–Cas9 is easier and cheaper to apply in engineering. A ZFN consists of a chain of zinc finger proteins fused with a bacterial nuclease to form a system able to make site-specific DSB. Zinc finger proteins provide site-specific targeting because they each recognize a 3–4 base pair DNA sequence. The nuclease usually used in ZFN system is *FokI*, which must dimerize to introduce a DSB [37,38].

The major disadvantage of ZFN technology is the probability of unwanted genes editing at off-target sites [41]. TALEN is structurally similar to ZFN. This system recognizes specific DNA base pairs via TAL effector, which is a natural protein secreted by bacteria *Xanthomonas* sp. The protein includes the C-terminal nuclear localization signal, the N-terminal translocation signal, the activation domain and the intermediate tandem repeat region, which is composed of many repeating sequence units arranged in series. *FokI* also needs dimerization from two TALENs to introduce DSBs [42,43,44]. The major disadvantage of TALEN technology is to induce mutation at off-target sites [41]. In contrast to them, Cas9 is an RNA-guided nuclease, and has sequence specificity largely due to Watson–Crick base pairing between the target DNA site and its gRNA, apart from direct interaction between Cas9 and PAM [14,45]. ZFNs and TALENs have been demonstrated to be efficient in genome editing, but new proteins must be created for them for each new DNA target site. Conversely, Cas9 proteins remain the same, irrespective of which DNA sequence is targeted, and only the short sequence of gRNA needs to be changed to redirect the site-specific cleavage. What is more, CRISPR–Cas9 is characterized by high efficiency due to the possibility of introducing modifications by direct insertion of RNAs encoding the Cas protein and gRNA. Furthermore, this system can lead to multiple gene modifications at the same time, because multiple gRNAs can be introduced simultaneously [46,47].

## 5. Epigenetic Regulation

Epigenetics refers to inherited changes in gene expression that do not include changes to the DNA sequence. Epigenetic mechanisms include DNA methylation and demethylation, histone posttranslational modifications, chromatin remodeling and non-coding RNA changes, and play important roles in various biological processes [48]. To perform the CRISPR/Cas9-mediated epigenome editing, the strategy is to fuse the nuclease-dead Cas9 (dCas9) with a transcription activator or repressor domain, known as an epigenetic effector (epieffector). DCas9 has no nuclease activity but acts as a DNA-binding domain. From many studies, it can be concluded that dCas9–epieffector fusion complex is an effective tool for epigenome editing [49]. In one in vitro study, the Krüppel-associated box (KRAB) effector domain was used. Thakore et al. targeted the dCas9–KRAB complex to the HS2 enhancer, a distal regulatory element, which coordinates the expression of multiple globin genes. Genome-wide analyses indicated that targeting dCas9–KRAB complex to HS2 specifically induces H3K9 tri-methylation (H3K9me3) at the enhancer and reduces chromatin availability to the enhancer. Epigenetic modification of HS2 silenced the expression of multiple globin genes [50]. In a different study, scientists demonstrated the utility of the CRISPR/Cas9 system for modulating methylation at specific CpG sites and inducing gene expression. They targeted murine Oct4 gene, which is transcriptionally blocked, due to hypermethylation in the promoter region in NIH3T3 cell line [51]. Oct4 is only expressed in stem cells and is responsible for self-renewal and the maintenance of the pluripotent state of stem cells. The hypomethylated promoter region of the Oct4 gene in stem cells is associated with its expression, while the hypermethylation state in differentiated cells leads to complete blockage of Oct4 [52]. To induce elicit site-specific demethylation at the Oct4 promoter region and its gene expression, CRISPR/Cas9 knock-in strategy was used. Genetically modified cells were used, in which the CpG dinucleotides of the promoter region were changed to non-methylated dinucleotides using CRISPR/Cas9-mediated knock-in system. As a consequence, Otc4 expression of all modified NIH3T3 cells was increased in comparison to wild-type NIH3T3 cells. What is more, the biggest increase in gene expression was observed in cells which had the CpG dinucleotides changes in the whole promoter region that included the CR1 region [51].

## 6. Gene Regulation

Besides the genes′ loss of function by formation of DSB, CRISPR–Cas9 can enhance or repress the expression of specific genes [53,54,55]. Research on *Escherichia coli* has been conducted. The activation of a gene can be achieved by a fusion of the inactive Cas9 with the transcriptional activation domain, whereas gene repression can be performed when dCas9, lacking cleaving activity, coexpresses with a gRNA, later generating a DNA recognition complex which can specifically interfere with transcriptional elongation, transcription factor binding or RNA polymerase binding. In practice, dCas9 loses the ability to break DNA but maintains its DNA binding activity and can inhibit gene expression by preventing initiation or elongation of transcription. In different study, *Streptococcus pyogenes* strains have been used to repress a β-galactosidase, resulting in 14-fold reduction in the activity of the enzyme. It has been shown that this system can be used to repress multiple target genes at the same time, and its effects are reversible [56,57,58]. SpCas9 is the best-characterized Cas9 ortholog, and thus is a suitable benchmark for defining the accuracy and efficiency of other Cas9s [59]. Nevertheless, there are other Cas9 proteins that have been studied and developed as tools for engineering. One of them is Cas9, adapted from *Staphylococcus aureus* (SaCas9), which has been developed for gene regulation and genome editing in mammalian cells. Research has shown that SpCas9 can be a high-specificity genome editing tool. SaCas9 and SpCas9 are amongst the most widely characterized Cas9 proteins and share ≈17% sequence identity and structural and mechanistic similarities. Both of them are widely used in different therapeutical and biotechnological applications [60,61]. However, SaCas9 is a multiple turnover enzyme, while SpCas9 cleaves a stoichiometric amount of DNA. What is more, SaCas9 does not have any detectable additional nuclease activity on cleaved DNA products, providing homogenous products [62].

## 7. Base Editing

Undoubtedly, the CRISPR–Cas9 method has gained considerable popularity in recent years and has been recognized as a powerful tool for modern medicine. Despite its many advantages, this tool is prone to errors, which can lead to undesirable mutations in the genome [63]. Base editing is a type of genome editing that provides direct, irreversible conversion of one base pair to another at the target genomic locus without requiring DSBs, HDR processes or a donor template [64]. Compared to previous genome editing strategies introducing point mutations, base editing can be more efficient with significantly fewer undesirable products, such as indels and translocations [65]. DNA base editors contain two components: a Cas enzyme for DNA binding and a single-stranded DNA modifying enzyme for targeting nucleotide modification. Two categories of DNA base editors have been presented: cytosine base editors (CBEs) and adenine base editors (ABEs). Thus, base editing can install four transition mutations (C→T, T→C, G→A and A→G) [66,67]. The first base editor (CBE1) converts cytosine to uracil by deaminating the exocyclic amine. Next, uracil is recognized by cell replication machinery as a thymine, which causes the C–G to T–A transition [68]. The system was designed by fusing a rat-derived cytosine deaminase apolipoprotein B mRNA editing enzyme catalytic subunit 1 (APOBEC1) to dCas9 [64]. Although CBE1 mediates efficient, targeted base editing in vitro, it is not effective in human cells. This decrease is mostly caused by cellular-mediated repair of the U–G intermediate in DNA by the base excision repair (BER) pathway. BER of U–G in DNA is initiated by uracil N-glycosylate (UNG), which identifies the U–G mismatch and cleaves the glycosidic bond between the uracil and the DNA deoxyribose backbone. It results in the reversion of the U–G intermediate created by CBE1 back editor to the C–G base pair [67]. To inhibit UNG, scientists fused uracil DNA glycosylase inhibitor (UGI) to the C-terminus of CBE1, thereby generating CBE2. It resulted in an increase in editing efficiency in human cells. Further attempts to improve editing efficiency led to designing the CBE3 by restoring histidine at position 840 (H840) in dCas9 and to create a base editor that uses Cas9 nickase (nCas9). This variant induces a nick in the G-containing strand of the U–G intermediate to bias cellular repair of the intermediate towards a U–A result, which is next converted to T–A during replication of DNA. This modification, in addition to increasing editing efficiency, has also increased the frequency of indels, but their rate is still much lower compared to frequency of indels induced by DSBs [64,65]. Subsequently, CBE4 was generated to improve editing efficiency, reducing indel formation and narrow the editing window. CBE4 was created by fusing an additional copy of UGI to the N-terminus of nCas9 with an optimized 27 bp linker [69]. ABEs act under a similar mechanism as CBE. The ABE–dCas9 complex binds to a target DNA sequence in gRNA-programmed manner; then deoxyadenosine deaminase domain catalyzes an adenine to inosine transition. In the DNA replication context, inosine is interpreted as guanine, and the A–T base pair can be replaced with a G-C base pair at the target site [67]. Liu and colleagues engineered *Escherichia coli* tRNA adenosine deaminase (TadA), which converts adenine to inosine in the single-stranded anticodon loop of tRNA. ABE1 were created by an antibiotic-resistance complementation in bacteria. To test TadA on a DNA target, *Escherichia coli* cells were equipped with TadA mutants and defective antibiotic resistance genes. To grow in the presence of antibiotic, a mutant TadA–dCas9 fusion had to convert a deoxyadenosine to deoxyinosine in the defective antibiotic resistance gene. ABE1 was generated by fusion of evolved TadA variant (TadA*) with the N-terminus of nCas9 by XTEN with the C-terminal of nCas9 fused with a nuclear localization signal (TadA*-XTEN-nCas9-NLS) [70,71]. To optimize ABE, a single chain heterodimer was engineered and consisted of a wild-type non catalytic TadA monomer and an evolved TadA monomer (TadA-TadA*). Different ABE revolutionary strategies, such as ABE7.10 and ABE8e, were developed to improve editing efficiency [64].

## 8. Prime Editing

Anzalone et al. from the Broad Institute of Harvard and MIT have developed an alternative method known as prime editing, which reduces the number of unintended errors [66,72]. Scientists showed that prime editing can install all 12 possible base-to-base conversions without the induction of DSBs in the target sequence or the requirement of donor DNA templates, because the prime editing does not rely on DSBs [73]. Prime editing includes prime editing guide RNA (pegRNA), which is longer than usual gRNA and Cas9(H840A) nickase fused to engineered reverse transcriptase (RT). The RT is an RNA-dependent DNA polymerase that uses the sequence from the pegRNA as a template. Three systems of prime editors (PE) have been constructed and tested in human cells. The first system (PE1) was made by a fusion of Cas9(H840A) nickase and wild type Moloney murine leukemia virus RT enzyme. In the case of the introduction of transversions point mutations, the PE1 efficiency was dependent on the primer binding site (PBS) length, and maximum efficiency was estimated at 0.7–5.5%. The application of the PE1 system led to low but detectable edits in the genome. In the second system, the thermostability, affinity and processivity of the DNA-RNA substrate was enhanced by introducing five specific mutations. Pentamutant RT fused with Cas9(H840A) nickase created the PE2 system. Introduction of two mutations (W313F, T306K) increased the RT thermostability and binding of RT with template-PSB complex, and also improved the efficiency of editing. Three other mutations (L603W, D200N, T330P) increased the number of introduced transversions and activity of RT at elevated temperatures. The PE2 contributed a 5.1-fold improvement in the efficiency of prime editing point mutation and performed targeted insertions and deletions more efficiently in comparison to PE1 [66]. Various studies have shown that nicks in an unmodified strand can increase the efficiency of basic editing systems in plant and animal cells [64,65,71]. To apply this strategy and improve prime editing, Anzalone et al. used a nickase (present in PE2) which was guided by sgRNA. As a consequence, this system was called PE3. What is more, PE3 was more efficient when sgRNA matched the newly-edited sequence introduced by the pegRNA, and this approach was labeled PE3b. The PE3 system can improve efficiency of editing about threefold in comparison to PE2 but with a higher probability of indels. Moreover, PE3b exhibits editing levels similar to PE3, while significantly reducing indel formation. Scientists have tested the prime editing capabilities to correct certain mutations, including mutations that cause diseases such as Tay–Sachs or sickle cell disease (SCD). In the case of Tay–Sachs disease, scientists created the mutant phenotype using the PE3 system in order to apply a 4 bp insertion into *HEXA*, with 31% efficiency and 0.8% indels. Reconstructing wild type phenotype using the PE3 system resulted in an editing efficiency of ≥20%, while using the PE3b system resulted in 33% efficiency and 0.32% indels. In regard to SCD, the PE3 system was used to apply the HBB E6V mutation to the HEK293T cell line. The experiments showed 44% efficiency and 4.8% indels. Anzalone et al. have used the PE3 system to apply a protective G•C-to-T•A transversion into PRION PROTEIN in the HEK293T cell line, introducing a G127V mutant allele, which confers resistance to prion disease in humans and mice. The most effective pegRNA with the PE3 system led to 53% installation of G127V and 1.7% indels [66].

## 9. CRISPR Applications—Functional Genome Screening

The CRISPR–Cas9 has a great potential in genome functional screening for identifying important genes in the biological processes of various biological models. It has been used in genome-wide, targeted loss-of-function screens as an alternative screening system to RNA interference (RNAi) [74]. Shalem et al. used a genome-scale CRISPR–Cas9 knockout (GeCKO) library to identify genes essential for cell viability in cancer and pluripotent stem cells. Next, they screened for genes whose loss is involved in resistance to vemurafenib (therapeutic for mutant protein kinase BRAF inhibition) in a melanoma model (A375 cell line) [75]. Different studies have identified host genes which are important for the intoxication of cells by diphtheria and anthrax toxins using GeCKO library. The results were also confirmed by functional validation [76]. The application of CRISPR screening in functional genomics is helping researchers to discover new gene functions and may change, e.g., cancer research in areas such as mechanism analysis and therapeutic exploration. In the future, CRISPR–Cas9 systems for functional genomic screening could be used in order to explore the molecular mechanisms of a variety of cellular functions. This will enable rapid drug identification with their therapeutic efficacy and allow one to use the potential of personalized medicine by combining genomics, disease phenotypes and therapeutic targets.

## 10. CRISPR Applications—Genetic Diseases

Despite significant advances in the identification of monogenic human disease genes, there are many challenges in alleviating these disorders. They are estimated to account for more than 10,000 diagnosed human diseases [77]. Among them are 5000–8000 monogenic diseases, defined as inherited conditions arising from mutations on a single gene. In the ClinVar database, more than 75,000 pathogenic genetic variants have been identified [66,78]. The therapeutic applications of CRISPR–Cas in model organisms are summarized in Table 3.

Leber congenital amaurosis (LCA) is a rare genetic eye disease manifesting severe vision loss at birth or infancy [83]. LCA10 is caused by bi-allelic loss-of-function mutations in the CEP290 gene. The manifestation of this mutation is severe retinal dystrophy and poor to no vision. The size of the CEP290 coding sequence (≈7.5 kilobases) exceeds adeno-associated virus (AAV) vector package capability. To overcome this limitation Maeder et al. developed EDIT-101, a candidate for a genome editing therapeutic, to eliminate the aberrant splice donor made by the IVS26 mutation in the CEP290 gene and restore normal CEP290 expression. This approach uses the identification of a pair of SaCas9 gRNAs specific to the human CEP290 target sequence. In vitro experiments were conducted on human cell lines (U2OS cell line, ARPE-19 cell line) and retinal explants and demonstrated the nuclease specificity and molecular mechanism of action. Subretinal EDIT-101 delivery in a humanized CEP290IVS26 knock-in mouse model showed that over 94% of the treated eyes achieved the therapeutic target editing level (10%) with an AAV dose of not less than 1 × 10^12^ vg/mL [79]. Duchenne muscular dystrophy (DMD) is an inherited musculoskeletal disease that exhibits clinical features of progressive muscle weakness in the early stages and pathological features of fibrosis and fat replacement, especially in the late stages of the disease. It is a recessive X-linked disease, occurring in 1 out of 3500 male births [84]. DMD mutations are frequently deletions of one or more exon in the dystrophin gene, which interfere with the reading frame of the gene and consequently lead to a complete loss of functional expression of dystrophin. Nelson et al. have developed an AAV-based strategy of DMD treatment in the *mdx* mouse model, using the unique multiplexing ability of CRISPR/Cas9 to excise the exon 23 from the dystrophin gene. Intramuscular injection resulted in 59% of transcripts with exon 23 deleted. The analyzes showed essential recovery of the dystrophin protein to ≈8% of the normal level and ≈67% of myofibers expressed dystrophin [80]. Sickle cell disease (SCD) is caused by a Glu- > Val mutation in β-globin subunit of hemoglobin leading to abnormal hemoglobin S. Re-expressing the paralogous γ-globin genes is a universal strategy for ameliorating β-globin disorders. Wu and colleagues applied CRISPR–Cas based cleavage of the GATA1 binding site of the erythroid enhancer. As a result, the expression of the erythroid γ-globin repressor *BCL11A* is decreased and the expression of γ-globin is increased. Editing of the *BCL11A* enhancer resulted in reduction in *BCL11A* transcript expression by 54.6%. This strategy is therapeutically feasible regarding producing stable fetal hemoglobin induction [81]. A group of scientists screened 14 Cas9/gRNA combinations for specific and efficient disruption of a nucleotide substitution that causes the dominant progressive hearing loss, DFNA36. As a model for DFNA36, they used *Beethoven* mice that carry a point mutation in *Tmc1*, a gene required for hearing, which encodes a pore-forming subunit of mechanosensory transduction channels in inner ear hair cells. They identified that SaCas9-KKH/gRNA can specifically recognize the mutant *Tmc1* but not the wild-type of Tmc1/TMC1 allele. The strategy was tested on *Beethoven* mice using AAV-mediated SaCas9-KKH delivery and showed strong therapeutic benefit in preventing deafness lasting up to one year after transduction. This discovery can provide a tool to efficiently and selectively disrupt the dominant single nucleotide mutation [82]. The diagnosis and treatment of monogenic diseases remains largely insufficient, and care is primarily palliative and focuses on disease management without addressing the underlying genetic defects. The use of the CRISPR–Cas9 system offers a new approach that can effectively help patients improve their comfort.

## 11. CRISPR Applications—Viral Infections

Most of the current human immunodeficiency virus (HIV), human papillomavirus (HPV), hepatitis B virus (HBV) and herpesvirus antiviral therapies do not provide a clinical cure—mainly due to the inability to remove the viral genome from the infected host cell because of a latent state, in which the viruses minimize their activity inside the host cell in order to avoid host immune surveillance. The latency-related life cycles of these viruses play the key role in the incurability of chronic infections. As a result, patients infected with these viruses have to take antiviral drugs for the rest of their lives. In this regard, the CRISPR–Cas9 system shows great promise as a therapy for chronic viral infections [85]. The CRISPR–Cas9 system has been demonstrated in the treatment of different viral infections. Zhen et al. have shown that CRISPR–Cas9 can be used to inhibit HBV replication and gene expression both in vitro (HepG2.2.15 cell line) and in vivo (BALB/c nude mice model). Inhibition was specific and sustained for 3 days after CRISPR–Cas9 administration. According to the results, this system may provide a simple, inexpensive and short-term process for mammalian genome modification [86]. Different studies have demonstrated that the CRISPR–Cas9 system may be potentially useful as a part of HIV virus treatment strategies. CRISPR–Cas9 delivered by lentivirus significantly decreased HIV-1 replication in infected primary CD4+ T cell cultures and considerably reduced viral load in ex vivo CD4+ T cell culture obtained from HIV-1 infected patients. A new therapeutic application may be eliminating HIV-1 DNA from CD4+ T cells, and CRISPR–Cas9 may be used as a novel and efficient platform for the cure of AIDS [87]. In another study, Wang et al. reported that the CRISPR–Cas9 system can be effective in latent viral infection treatment. Burkitt’s lymphoma cell line with latent Epstein–Barr virus (EBV) infection was used as a natural model. They demonstrated that about 25% of treated cells were relieved of EBV and no viral DNA was detected. Another 50% of cells showed considerable EBV load decrease in comparison to the untreated sample [88]. The use of CRISP–Cas9 as an antiviral strategy is a promising prospect and various in vitro and in vivo studies have given rise to potential clinical applications for humans. By combining CRISPR–Cas9 with more sophisticated in vivo and ex vivo models, and ultimately clinical trials, it will be possible to determine the efficacy and target safety, and potentially treatment or prevention methods for pathogenic viral infections in humans.

## 12. CRISPR Applications—Antibiotic Resistant Bacteria

The widespread and often unjustified use of antibiotics in the public health system and in agriculture over the last seven decades led to the development of bacterial antibiotic resistance mechanisms. Bacterial populations have become resistant to the antimicrobials through genetic changes and additions. Various strategies have been implemented against antibiotic-resistant bacteria, including the production of new antibiotics, using the bacteriophages or peptides or enzymes of natural or synthetic origin that specifically target the bacterial genomes or their functional proteins. However, the effects of these strategies are not satisfactory, and it is predicted that drug-resistant pathogens will cause 10 million deaths annually by 2050 [89,90]. Research indicates that the CRISPR–Cas system can be effectively used to prevent, control and combat the antibiotic-resistant bacteria. Methicillin-resistant *Staphylococcus aureus* (MRSA) is a human pathogen which is resistant to β-lactam antibiotics such as penicillin, methicillin and oxacillin. Wang et al. have designed two CRISPR–dCas9 systems to repress antibiotic resistance in MRSA by targeting a different site on the *mecA* methicillin resistance gene. Results of the experiments have shown a 77% decrease in gene expression in CRISPR-treated samples; however, 77% was not sufficient to make MRSA clinically susceptible to β-lactam antibiotics. The CRISPR–dCas9 system applied in these experiments did not kill the bacteria, which makes it a viable option for treatment against antibiotic-resistant bacteria [91]. In a different study, Kang et al. introduced a nonviral delivery method for CRISPR (Cr-Nanocomplex), based on a polymer-derivatized Cas9 protein and sgRNA nanocomplex targeting *mecA*—a major antibiotic resistance gene involved in MRSA. Recombinant SpCas9 was covalently modified with a cationic polymer, known as branched polyethyleneimine (bPEI), as the carrier for packaging sgRNA and boosting their delivery to bacteria. This modification enabled the formation of nanosized complexes during mixing with sgRNA. It has been shown that Cas9 conjugated with bPEI can be taken up into the bacteria better than native Cas9 simply (noncovalently) mixed with bPEI and native Cas9 mixed with lipofectamine as a carrier, which did not show any sign of uptake. The cultured MRSA strains which were treated with Cr-Nanocomplex were not able to grow in agar media including oxacillin in the dose 6 µg/mL, whereas the strains that were not treated could grow in the media. Results have shown that MRSA treatment with the Cr-Nanocomplex could decrease the growth (32% decrease) in contrast to treatment with the Cas9-bPEI without sgRNA as the control [92,93]. The use of CRISPR–Cas9 to specifically remove resistance genes can be a powerful tool to counteract antibiotic resistance and could be a part of the solution to keep antibiotics working. The current approach is to control the composition of microbial community instead of using CRISPR–Cas9 as a conventional broad-spectrum antibiotic, which can potentially target only antibiotic-resistant bacteria, while preserving commensal ones in the microbiota.

## 13. CRISPR Applications—Cancer Therapy

Over the past few decades, various anti-cancer therapies have been introduced, including immunotherapy, chemotherapy, targeted antibodies, hormone therapy, targeted drug therapy and surgery [94]. Despite significant advances in treatment, cancer causes over nine million deaths worldwide each year. These data demonstrate the need for continuous understanding of the biological characteristics of cancer cells and the molecular mechanisms of disease. In this regard, genome editing offers enormous opportunities for increasing knowledge of cancer biology, for developing new preclinical models and for progress in more efficient and targeted cancer cell elimination strategies [95]. Nowadays, numerous clinical trials use CRISPR–Cas9 system in therapies of multiple types of cancer. Most of them are focused on genetically engineered T-cells and cancer immunotherapy and target specific genes in the cancer cells. The lack of a safe and efficient delivery method which can be applied in clinical trials is one of the major problems related with direct targeting in cancer. What is more, tumor heterogenicity can be another problem in treatment, because tumors usually consist of different subclones [96,97]. Koo et al. have demonstrated that delivery of EGFR mutation-specific CRISPR–Cas9 through adenovirus (Ad) vector into EGFR mutant-bearing tumors resulted in the cleavage and disruption of the mutant EGFR alleles with high precision. Disruption of the EGFR mutation (L858R) in H195 tumors resulted in cancer cell death and a significant reduction of tumor size in vivo [98]. Cancer immunotherapy requires genetic engineering of immune cells of a patient to recognize and destroy cancer cells. CRISPR–Cas9 system can simplify the generation of therapeutic cell products due to the flexibility of this method [40]. In one study, the CRISPR–Cas9 has been used to generate chimeric antigen receptor (CAR) T cells, which can recognize specific antigens on cancer cells. Scientists infused autologous T cells transduced with a CD19-directed chimeric antigen receptor lentiviral vector in patients with refractory or relapsed acute lymphoblastic leukemia (ALL). Therapy using CAR-T cells against CD19 was effective for treatment of ALL and was related to a high remission rate also in patients for whom stem-cell transplantation was unsuccessful [99]. Additional applications of CRISPR–Cas9 in the treatment of various cancers are listed in Table 4.

Editing efficiency is one of the challenges of CRISPR–Cas9 method in cancer therapy. Editing efficiency improvements associated with reduced off-target effects are significant to achieving better general therapeutic efficacy. The effectiveness of DSB repair via HDR and NHEJ varies markedly in different cell types and cell states. Under low editing efficiency or a lack of adaptability conditions of edited cells compared to unedited cells, the therapeutic effect is not as expected [107,108]. The immune response induced by Cas9 protein itself can be also a problem. It is possible that this is due to the presence of certain peptides in Cas9 that may act as MHC-binding epitopes. It should be remembered that Cas9 is a protein of bacterial origin and can have an immunogenic effect in mammals [109]. CRISPR–Cas9 appears to be crucial in cancer research, especially in the studies concerning involved in carcinogenesis individual genes function. However, there are still many questions about the approaches in which CRISPR–Cas9 might be utilized in cancer research and treatment. It will still be necessary to optimize CRISPR–Cas9′s safety, specificity and efficacy before its usage in clinical practice.

## 14. CRISPR—Limitations

There are potential limitations associated with the use of CRISPR–Cas9 technology. In research using RNA-targeted gene editing technology based on CIRSPR-Cas9, all off-target effects should be carefully investigated. The consequence of the persistence of genetic drift in a population could be the off-target mutations that will persist in each generation. Moreover, effect and number of mutations can increase as the generations progress [110]. The dispersion of a feature of gene driving can be difficult to control. What is more, destroying the whole population targeted by gene drive can have severe effects in the ecosystem’s balance. Another issue is the potential transfer of genes to other species in the environment. The consequence of this can be the transmission of negative features to related organisms [111,112]. By comparison, off-target mutations are more common in human cells than in zebrafish or mice [113,114]. Genetic mosaicism in founders can be one of the results of CRISPR–Cas9 mediated gene editing in embryos, especially in the generation of transgenic and knockout animal models. CRISPR–Cas9 components are usually injected as RNA, DNA or protein molecules directly to fertilized zygotes [114,115,116]. This is undesirable in the majority of applications due to the formation of false-positive genotyping results. For example, the founder mouse can show a homozygous deletion of candidate based on tail DNA genotyping; however, it may never transmit the deletion allele to the offspring [117]. Genetic mosaicism makes phenotype analysis difficult in F0 animals, especially when carrying-out HDR-mediated targeted DNA insertions. Many F0 mosaic embryos should be screened to receive homogenous germline transmissions with the desired mutations [118,119]. Tu et al. have reported that tagging Cas9 with ubiquitin-proteasomal degradation signals can simplify the Cas9 degradation, thereby reducing mosaic mutations and increasing its ability of genome modification in non-human primate embryos [120].

## 15. CRISPR—Ethical Considerations

Significant advances in CRISPR–Cas9 technology have introduced challenges in regulating its safe and ethical use. Several groups of scientists have used CRISPR–Cas9 to edit genes in human embryos, even though it was always done in non-viable, triploid zygotes [121,122]. This type of research was carried out to investigate the specificity and accuracy of the CRISPR–Cas9 system [123]. This led to a debate in the scientific community on how to ethically and responsibly use gene editing methods in a way that does not hinder the benefits of research results and discoveries [124]. In November 2018, the U.S. National Academy of Sciences, the U.S. National Academy of Medicine, the Royal Society of the United Kingdom and the Academy of Sciences of Hong Kong convened the Second International Summit on Human Genome Editing. Over 500 researchers, policymakers, ethicists, representatives from medical and scientific academies, patient groups’ representatives and others attended the summit. During the event, the potential benefits and risks of editing the human genome; cultural and ethical perspectives; regulatory and policy issues; and public outreach were debated. The Second International Summit followed the First International Summit on Human Genome Editing in December 2015. In a statement published after a previous summit, the committee noted that intensive basic and preclinical study on genome editing was necessary and this type of study should be subject to proper legal and ethical rules concerning both somatic (nonheritable) and germline (heritable) human genome editing. Despite the fact of many precedents, the general acceptance of modifying single person’s somatic DNA is more tolerable than a germline, which could be passed to offspring. The first case is significantly beneficial in treatment of hemophilia, HIV, cancer, Alzheimer’s disease and any other novel diseases [125,126]. In the second case, germline gene modification lacks societal consensus, and some countries even outlaw this practice. What is more, genome editing of germline cells that could be passed on to the next generations as a part of the human gene pool seems irresponsible until the safety concerns are resolved. It should be mentioned that this aspect should consider the prevention of the transmission of genetic variants associated with illness, decreasing the probability of diseases developing and enhancing human capabilities. In first regard, genome editing will give a parents another option instead to pass to offspring the genetic variants responsible for, e.g., Huntington’s disease or not having biologically related children at all. The second option, will allow one to inactivate the particular genes resulting in lowering blood pressure or cholesterol level. The third and the most controversial, will allow one to enhance human functions and create superhumans by increasing, e.g., strength [73]. At the second summit, it was found that germline genome editing could be morally permitted in certain circumstances, but there are no such circumstances in the world. In addition, one opinion was that the benefits and risks of the genome editing of germline cells are still not clear enough to allow germline genome editing to continue [127,128].

## 16. Conclusions

The intensive development of the CRISPR–Cas9 system is creating significantly beneficial results in modern science. It allows for transcriptional regulation, genomic modifications and epigenetic editing with simplicity and high efficiency. The CRISPR–Cas9 system has been successfully used to edit the genomes of a broad range of species, such as *Caenorhabditis elegans* [129], *Drosophila* [130,131], zebrafish [132], *Bombyx mori* [133] and humans [134]. The CRISPR–Cas9 technology as a powerful, inexpensive and quick to design genome editing tool has been applied in many fields, ranging from basic biology to cancer therapy [135,136]. Research on the use of this system in the treatment of various diseases is ongoing. It has been found that CRISPR technologies play a major role in the metadata revolution and can help to understand specific gene functions thanks to their ability to precisely analyze genetic networks. The knowledge thus obtained can be used for personalized human disease treatment [137]. In addition to the health sector, CRISPR–Cas9 has great potential to affect agriculture and is used to expedite livestock and crop breeding [138]. On the other hand, there are numerous controversies related to CRISPR, including off-target effects, the immunogenicity of Cas9 nucleases and carcinogenic effects of CRISPR components, which require exhaustive analysis and scientific explanations [139].

## Figures and Tables

**Table 1:** Overview of CRISPR–Cas classification and characteristics [17,18,19,20].

Class	Type	Effector Module	Nuclease Domain	RracrRNA	Cleaving Target
I	I	Multiple Cas proteins—Cas3 (sometimes fused to Cas2), Cas5–Cas8, Cas10 and Cas11 in different combinations, depending on the type and subtype	Histidine-aspartate domain (HD) fused to Cas3	No	DNA
	III		HD fused to Cas10	No	
	IV		Unknown	No	
II	II	Cas9 protein	RuvC and HNH	Yes	
	V	Cas12a (Cpf1)/Cas12b/Cas12c	RuvC and Nuc	Cpf1-No	
	VI	Cas13a/Cas13b/Cas13c	Higher eukaryotes and prokaryotes nucleotide-binding (HEPN) domains	No	RNA

**Table 2:** Systematic comparison of the three genome editing tools: ZFN, TALEN, CRISPR–Cas9 [38,39,40,41].

	Zinc-Finger Nuclease ZFN	Transcription Activator-Like Effector Nuclease TALEN	Clustered Regularly Interspaced Palindromic Repeats-CRISPR-Associated-9 (CRISPR–Cas9)
Construction	Protein engineering for every single target	Protein engineering for every single target	20-Nucleotide sequence of single-guide RNA (sgRNA)
Target sequence recognition	Zinc fingers protein, protein-DNA interactions	Repeat variable diresidues (RVDs) repeats, protein-DNA interactions	sgRNA, RNA-DNA interactions
Endonuclease	FokI	FokI	Cas9 and its different variants
Endonuclease construction	3–4 Zinc fingers domains	8–31 RVD repeats	sgRNA synthesis or cloning
Delivery	Two ZFNs around the target sequence	Two TALENs around the target sequence are required	sgRNA complementary to the target sequence with Cas9 protein
DNA sequence recognition size	(9 or 12 bp) × 2	(8–31 bp) × 2	17–20 bp + NGG × 1
Targeting efficiency	Low	Moderate	High
Affordability	Resource intensive and time consuming	Affordable but time consuming	Highly affordable and rapid

**Table 3:** CRISPR–Cas applications in selected monogenic diseases.

Disease	Target	Animal Model	Delivery System	Strategy	References
Leber congenital amaurosis type 10 (LCA10)	*CEP290*	HuCEP290 IVS26 KI mouse eye	Adeno-Associated Virus (AAV); (subretinal injection)	Non-homologous end joining (NHEJ) mediated aberrant splicing	[79]
Duchenne muscular dystrophy (DMD)	*Dmd*	mdx mice muscle	AAV; intramuscular injection	NHEJ mediated mutant exon 23 skipping	[80]
Sickle cell disease (SCD)	*BCL11A* erythroid enhancer	CD34+ human hematopoietic stem/progenitor cells (HSPCs) from sickle cell disease patient	Ribonucleoprotein (RNP); electroporation	NHEJ mediated enhancer disruption	[81]
Genetic Deafness	*Tmc1*	Beethoven (Bth) mouse ear	AAV; Inner ear injections	NHEJ mediated mutant Tmc allele disruption	[82]

**Table 4:** CRISPR–Cas9 applications in the different types of cancer treatment.

Cancer Type	Target	Type of Study	Vector	CRISPR Results	Reference
Lung cancer	*Pten*	In vitro (A549 and NCI-H460 cell lines)	Plasmid	*PTEN* knockout	[100]
	*EGFR*	In vitro (NCI-H1975 and NCI-H1650 cell lines)	Lentiviral	*EGFR* knockout	[101]
Breast cancer	*BRCA1, BRCA1m*	In vitro (MDA-MB-231 and MDA-MB-436 cell lines)	Plasmid	PARP1 knockout	[102]
	*APOBEC3G*	In vitro (MCF10A and HCC1806 cell lines)	Plasmid	Knockout of both alleles	[103]
Liver cancer	*Pten, p53*	In vivo (mice model)	Plasmid	Knockout of both alleles	[104]
Colorectal cancer	*KRAS*	In vitro (SW-480 and 293T cell lines)	Polymer	Knockout of both alleles	[105]
Prostate cancer	*p53*	In vitro (PC-3 cell line)	Plasmid	Knockout of both alleles	[106]
